# Genetic characterization of non-5q proximal spinal muscular atrophy in a French cohort: the place of whole exome sequencing

**DOI:** 10.1038/s41431-023-01407-8

**Published:** 2023-06-19

**Authors:** Julian Theuriet, Gorka Fernandez-Eulate, Philippe Latour, Tanya Stojkovic, Marion Masingue, Léo Vidoni, Emilien Bernard, Arnaud Jacquier, Laurent Schaeffer, Emmanuelle Salort-Campana, Jean-Baptiste Chanson, Aleksandra Nadaj Pakleza, Anne-Laure Kaminsky, Juliette Svahn, Véronique Manel, Françoise Bouhour, Antoine Pegat

**Affiliations:** 1 Hôpital Neurologique Pierre Wertheimer, Service d’électroneuromyographie et de Pathologies Neuromusculaires, Hospices Civils de Lyon, Groupement Est, Bron, France; 2 Pathophysiology and Genetics of Neuron and Muscle, CNRS UMR 5261, INSERM U1315, Université Lyon1, Faculté de Médecine Lyon Est, Lyon, France; 3 Nord/Est/Ile-De-France Neuromuscular Reference Center, Institut de Myologie, Hôpital Pitié-Salpêtrière, Assistance Publique des Hôpitaux de Paris, Paris, France; 4 Unité Fonctionnelle de Neurogénétique Moléculaire, Hospices Civils de Lyon, Groupement Est, Bron, France; 5 Hôpital Neurologique Pierre-Wertheimer, Service de Neurologie, Troubles du Mouvement et Pathologies Neuromusculaires, Hospices Civils de Lyon, Groupement Est, Bron, France; 6 Centre de Biotechnologie Cellulaire, CBC Biotec, Hospices Civils de Lyon, Groupement Est, Bron, France; 7 Hôpital de la Timone, Maladies Neuromusculaires et SMA, Assistance Publique des Hôpitaux de Marseille, Marseille, France; 8 Centre de Référence des Maladies Neuromusculaires Nord/Est/Ile-de-France, Service de Neurologie, Hôpitaux Universitaires de Strasbourg, Strasbourg, France; 9 Service de Neurologie, Centre Référent des Maladies Neuromusculaires Rares, CHU de Saint Etienne, Saint-Etienne, France; 10 Hôpital Femme Mère Enfant, Service de Neuropédiatrie, Hospices Civils de Lyon, Groupement Est, Bron, France

**Keywords:** Motor neuron disease, Peripheral neuropathies

## Abstract

Proximal spinal muscular atrophy (SMA) is defined by a degeneration of the anterior horn cells resulting in muscle weakness predominantly in the proximal lower limbs. While most patients carry a biallelic deletion in the *SMN1* gene (localized in chromosome 5q), little is known regarding patients without *SMN1*-mutation, and a genetic diagnosis is not always possible. Here, we report a cohort of 24 French patients with non-5q proximal SMA from five neuromuscular centers who all, except two, had next-generation sequencing (NGS) gene panel, followed by whole exome sequencing (WES) if gene panel showed a negative result. The two remaining patients benefited directly from WES or whole genome sequencing (WGS). A total of ten patients with causative variants were identified, nine of whom were index cases (9/23 families = 39%). Eight variants were identified by gene panel: five variants in *DYNC1H1*, and three in *BICD2*. Compound heterozygous causative variants in *ASAH1* were identified directly by WES, and one variant in *DYNC1H1* was identified directly by WGS. No causative variant was found using WES in patients with a previous panel with negative results (14 cases). We thus recommend using primarily NGS panels in patients with non-5q-SMA and using WES, especially when several members of the same family are affected and/or when trio analyses are possible, or WGS as second-line testing if available.

## Introduction

Proximal spinal muscular atrophy (SMA) is defined by degeneration of the anterior horn cells resulting in weakness and muscle atrophy, predominantly affecting the proximal lower limbs. Several forms of SMA have been described in association with different gene mutations [1].

The most common form, representing 95% of all cases, is characterized by a recessive deletion, and more rarely by a deletion associated with a point mutation, in the 5q13 survival motor neuron (*SMN1*) gene [1]. In patients with an *SMN1* biallelic deletion, the SMN protein production is consequently abnormal and insufficient [1]. The SMN protein plays a role in the spliceosomal small nuclear ribonuclear protein biogenesis and pre-mRNA splicing. The understanding of these genetic abnormalities has led to the development of effective therapies, such as the antisense oligonucleotide (ASO) therapies [2].

Little is known however, about forms of the disease without the *SMN1* deletion, even though the number of causative genes responsible for other rare forms of proximal SMA has increased over the past few years (especially *CHCHD10, TRPV4, DYNC1H1, BICD2, HEXA*, and *HEXB*) [3, 4]. Moreover, SMA is a rare pathology with an incidence of 1 in 11 000 live births for the most common form. Consequently, forms of the disease without *SMN1* deletion represent a diagnostic and therapeutic challenge [5].

The primary aim of the present study was to determine the contribution of whole exome sequencing (WES) in SMA patients without *SMN1* homozygous deletion (referred to as non-5q-SMA), using a retrospective French cohort of 24 patients. The secondary aim was to determine predictive factors of a positive genetic test.

## Methods

### Study design and population

This retrospective, observational, and multicenter study included patients who consulted in the neurology departments of specialized neuromuscular centers in Lyon, Paris, Marseille, Strasbourg, and Saint-Etienne between 1983 and 2021 for proximal muscle weakness, predominantly affecting the lower limbs, with motor axonal neuropathy on nerve conduction study and electromyography (EMG). The latter was defined by the presence of neurogenic motor unit potentials and/or a reduction of compound motor action potentials (CMAP), with a normal or <30% reduction of sensory nerve action potentials (SNAP). After exclusion of other diagnoses, and in the absence of a homozygous deletion in the *SMN1* gene, all patients had a diagnosis of non-5q proximal SMA. All clinical data were collected anonymously from the study units’ medical files. All patients provided written informed consent for genetic tests and use of their data for research purposes. All procedures involving patients performed in this study were carried out in accordance with the ethical standards of the Hospices Civils de Lyon (ethics approval #22_846) and with the 1964 Helsinki declaration.

### Clinical, laboratory, and electrophysiological data

The demographic data collected were age at onset, family history of SMA, and parents’ consanguinity. The clinical data of interest included the predominant site of muscle weakness in the lower limbs, the presence of muscle weakness in upper limbs, and if present, the weakness was classified as proximal (predominantly involving deltoid, biceps, or triceps) or distal (predominantly involving flexor digitorum, extensor digitorum, interosseous, or abductor pollicis brevis). Other clinical data were notified: axial muscle weakness (defined as a muscle weakness ≤ 4/5 according to the Medical Research Council (MRC) scale on neck flexion, and/or as an impossibility to rise from a lying position without hands), osteoarticular deformities (scoliosis, *pes cavus*, *pes planus, pes equinovarus, pes planovalgus*, clubfoot and/or articular retractions), tremor (classified as a postural or resting tremor), pyramidal signs (brisk reflexes, Babinski or Hoffman’s signs, or spasticity), and scapular winging. Then, EMG were performed in each specialized neuromuscular center by trained neurologists. Patients were classified as having fibrillations and/or positive sharp waves in the first EMG or not. Creatine kinase (CK) was considered as elevated above 200 UI/L.

### Genetic analyses

Overall, 22 of the 24 patients first benefited from the same next-generation sequencing (NGS) gene panel analysis, composed of 103 genes involved in hereditary peripheral neuropathy using Capture Roche KAPA HyperCap v3.0 (Pleasanton, CA, USA), and sequenced on Illumina^®^ NextSeq500 (San Diego, CA, USA; Supplementary Table 1). In patients with no causative variant identified using the gene panel analysis, WES were performed using Capture Roche MedExome (Pleasanton, CA, USA), and sequenced on Illumina^®^ NextSeq500 as previously described [6]. Reads were aligned to the human reference genome hg37/GRCh37 (Genome Reference Consortium Human GRCh37). The following filters were applied: heterozygosity threshold of 0.20, read depth >14 for homozygous variants and >7 for each allele for heterozygous variants, frequency in 1000 Genomes database <0.01, heterozygous occurrence <250 in ExAc database, maximum occurrence of 6/12 in the run, and introns usually covered at +/−60 base pairs (bp). A total of 16,582 variants remained after the application of these filters. Genes known to be associated with non-5q proximal SMA [7, 8] or amyotrophic lateral sclerosis (ALS) [9], another disease affecting motoneurons, were individually checked (Supplementary Table 2). The two other patients benefited either directly from WES (patient 10) or from WGS (patient 4) after a negative panel analysis of genes associated with myopathy (initial suspicion of myopathy). Finally, identified variants were searched in the ClinVar and gnomAD databases, and their pathogenicity was evaluated according to the American College of Medical Genetics and Genomics (ACMG) guidelines, and in silico analyses of variants were performed using three softwares (PolyPhen2, SIFT, and MutationTaster).

### Statistical analyses

Data were analyzed using Excel (Microsoft) and R version 4.2.1. Qualitative variables were described using proportions for descriptive analyses, and quantitative data were described using medians and interquartile ranges. Due to the small sample sizes and non-normal distribution, non-parametric tests were used to compare patients with and without an identified variant. Fisher’s exact test was used to compare proportions and the Mann–Whitney test was used for median comparisons. For a given variable, a statistic test was performed only if the event was observed more than four times in the cohort, to allow Fisher’s exact test to detect a significant difference between groups. Correction for multiple testing was made using the Benjamini and Hochberg method.

## Results

**Fig. 1:**
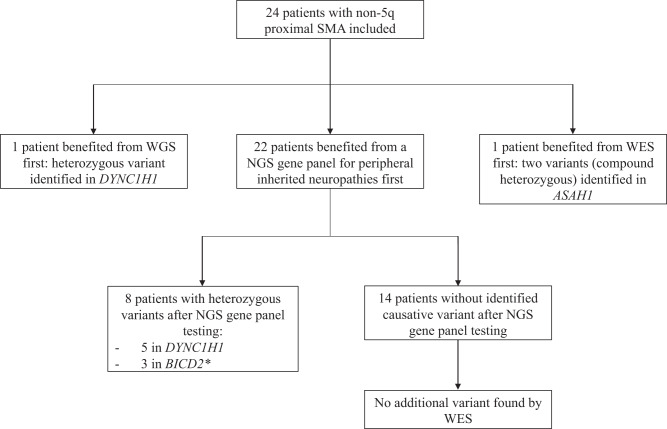
Diagram of genetic testing and identified variants. SMA spinal muscular atrophy, WES whole exome sequencing, WGS whole genome sequencing, NGS next-generation sequencing. *The patient with the c.380A>G variant in *BICD2*, classified as VUS, also benefited from WES which did not find an additional causative variant.

The study population was composed of 24 patients from 23 families (patients 2 and 3 were mother and son) affected by non-5q proximal SMA, from five French centers specialized in neuromuscular disorders (11 from Paris, nine from Lyon, two from Strasbourg, one from Marseille, and one from Saint-Etienne); 18 patients were female. Consanguinity of the patients’ parents was never reported. The description of the genetic tests and the corresponding identified variants are available in Fig. 1. Overall, a variant was identified in 9 out of 23 index cases (39%). For the 22 patients who first benefited from an NGS gene panel analysis of hereditary peripheral neuropathy, a causative variant was identified in eight of the patients belonging to seven families (7/22 families = 32%; Table 1). All variants identified by the NGS panel were heterozygous. Four disease-causing variants were identified in *DYNC1H1* (NM_001376.5), affecting five patients (from four families). The variants c.1792C>T and c.2327C>T were already classified as pathogenic on ClinVar and gnomAD databases, and were classified as likely-pathogenic according to the ACMG criteria [10, 11]. Two other variants detected in *DYNC1H1* were absent from the ClinVar and gnomAD databases (c.596A>C and c.1427T>C) [10, 11]. The c.596A>C variant in DYNC1H1 occurred de novo. These two variants (c.596A>C and c.1427T>C) were also classified as likely-pathogenic according to the ACMG guidelines. Three variants were identified in *BICD2* (NM_001003800.2), with one variant not reported in the ClinVar and gnomAD databases (c.380A>G), while the two others (c.1922T>C and c.2042C>T) were classified as variants of unknown significance (VUS) [10, 11]. The c.1922T>C variant in BICD2 occurred de novo. Herein, the c.1922T>C and c.2042C>T variants were classified as likely-pathogenic while the c.380A>G was classified as VUS according to the ACMG criteria. The three previously unreported variants were predicted to be pathogenic by the three in silico algorithms used (Supplementary Table 3). A WES was performed in the 14 patients with no identified causative variant after the NGS panel, and no additional variant was identified. Particularly, no disease-related variant was identified in *SMN1*. Patient 9, harboring the c.380A>G variant in *BICD2*, specifically benefited from a WES because of the VUS classification according to the ACMG criteria, and no additional SMA-related variant was identified. Patient 22 was the only male with a negative NGS panel and WES, and thus benefited from specific genetic testing for Kennedy disease (AR gene), which was also negative.

**Table 1:** List of identified variants.

Gene, Variant	Zygosity	ClinVar pathogenicity (dbSNP reference number) (10)	Method used to detect variant	ACMG classification (12)	Number of index cases
*DYNC1H1* (NM_001376.5)					5
c.1792C>T (p.Arg598Cys)	Heterozygous	Pathogenic (rs587780564)	NGS panel	4 (PM1, PM2, PP3, PP5)	1
c.751C>T (p.Arg251Cys)	Heterozygous	Conflicting interpretations (rs879253979)	WGS	4 (PS2, PM1, PP2, PP3)	1
c.2327C>T (p.Pro776Leu)	Heterozygous	Pathogenic (rs1057518083)	NGS panel	4 (PM1, PM2, PP3, PP5)	1
c.596A>C (p.Asn199Thr)	Heterozygous	Not reported	NGS panel	4 (PS2, PM2, PP3)	1
c.1427T>C (p.Leu476Pro)	Heterozygous	Not reported	NGS panel	4 (PM1, PM2, PP3, PP4)	1
*BICD2* (NM_001003800.2)					3
c.2042C>T (p.Ser681Leu)	Heterozygous	VUS (rs1853375829)	NGS panel	4 (PM1, PM2, PP3, PP4)	1
c.1922T>C (p.Leu641Pro)	Heterozygous	VUS (rs1554705383)	NGS panel	4 (PS2, PM2, PP3)	1
c.380A>G (p.Glu127Arg)	Heterozygous	Not reported	NGS panel	3 (PM1, PM2, PP3, BP4)	1
*ASAH1* (NM_177924.3)					1
c.77C>G (p.Pro26Arg) and c.125+1G>A	Compound heterozygous	Pathogenic (rs886039750, rs1588999312)	WES	5 (PS3, PM1, PM2, PM3, PP5, BP4) 5 (PS3, PM2, PM3, PP5)	1
Total					9

*VUS* variant of unknown significance, *NGS* next-generation sequencing, *WGS* whole genome sequencing, *WES* whole exome sequencing, *ACMG* American college of medical genetics and genomics.

For patient 10, a WES was performed first, and biallelic variants (c.77C>G and c.125 + 1G>A) were found in *ASAH1* (NM_177924.3), classified as pathogenic according to the ACMG guidelines. Analyses in the family confirmed the bi-parental segregation of the *ASAH1* variants [12]. Patient 4 benefited from a WGS first, which identified a c.751C>T variant in *DYNC1H1*, the pathogenicity of which had conflicting interpretations in the ClinVar database. It occurred de novo and was classified herein as likely-pathogenic according to the ACMG criteria. The Sanger sequencings of the de novo and/or not previously reported variants, which were identified through the gene panel, are available in Supplementary Fig. 1.

Then, predictive factors for informative genetic testing were determined by comparing two groups: one (*n* = 10) with an identified variant, and the other one (*n* = 14) without an identified variant. After correction for multiple testing, only the age at onset remained significantly different between the two groups (*p*-value = 0.01), with a median age at onset of 3.5 years and 27.5 years in patients with and without identified variants, respectively (Table 2). Indeed, 9/10 (90%) patients with an identified causative variant had a disease onset before the age of 15 years. In patients with disease onset before the age of 18 years, a genetic diagnosis was possible in 9/12 patients (75%; Table 3). Interestingly, osteoarticular deformities tended to be more frequent in mutated patients (90% vs 35.7%, *p*-value = 0.07). In the group with an identified variant there was a tendency towards an increased frequency of family history of SMA (40% vs 7.1%, *p*-value = 0.26) and a pure lower limb involvement (90% vs 50%, *p*-value = 0.21). The clinical pattern of weakness in the lower limbs, the presence of tremor, axial muscle weakness, scapular winging, and pyramidal signs were not significantly different between groups. Regarding complementary exams, neither the CK elevation in blood samples nor the presence of decreased CMAP or fibrillations was significantly different between patients with and without identified variants (Table 2).

**Table 2:** Comparison of demographic and clinical characteristics between patients with and without causative variants identified by genetic testing.

	Patients with identified variants	Patients without identified variants	Adjusted *p*-value^a^
Number of patients	10	14	
Female sex	5 (50)	13 (92.9)	0.18
Age at onset, years, median [IQR]	3.5 [1.125–10.75]	27.5 [22–34.5]	0.01
Family history of SMA	4 (40)	1 (7.1)	0.26
Parents’ consanguinity	0 (0)	0 (0)	
Lower limb weakness			1
Global	3 (30)	5 (35.7)	
Quadriceps	4 (40)	4 (28.6)	
Psoas	3 (30)	5 (35.7)	
Upper limb weakness	1 (10)	7 (50)	0.21
Axial muscle weakness	3 (30)	6 (42.9)	0.83
Osteoarticular deformities	9 (90)	5 (35.7)	0.07
Tremor	3 (30)	4 (28.6)	1
Pyramidal signs	1 (10)	2 (14.3)	
Scapular winging	3 (30)	1 (7.1)	
CK elevation	2 (25)	8 (61)	0.33
Decreased CMAP	3 (30)	2 (14.3)	0.84
Fibrillations or positive sharp waves on 1^st^ EMG	1 (10)	5 (35.7)	0.53

Data are expressed as number and percentage unless otherwise specified.

A statistic test was performed only if the event was observed more than four times in the cohort.

*SMA* spinal muscular atrophy, *CK* creatine kinase, *CMAP* compound muscle action potential, *EMG* needle electromyography.

^a^Using Benjamini and Hochberg method.

**Table 3:** Clinical and paraclinical characteristics of the cohort of non-5q proximal SMA patients.

Number	Age at onset (years)/sex/age at exam (years)	Family history of SMA (relationship)	Muscle predominance of lower limb weakness	Upper limb weakness (prox., dist. or PD)	Axial muscle weakness	Osteoarticular deformities (type)	Tremor (R or P)	Pyramidal signs	Scapular winging	Walking aid at last exam (age at last exam in years)	CK elevation	Fibrillations or positive sharp waves on 1^st^ EMG (years after symptom onset)	Genes and variants
1	1/H/23	+ (brother)	Psoas	−	−	+ (club foot*, pes cavus*)	−	−	−	− (32)	+	− (22)	*DYNC1H1* c.1427T>C
2	1.5/M/11.5	+ (mother)	Quadriceps	−	+	+ (scoliosis)	−	−	+	− (11.5)	−	− (10)	*DYNC1H1* c.1792C>T
3	5/F/35	+ (son)	Global	−	−	−	+ (P)	−	−	− (35)	NA	− (30)	*DYNC1H1* c.1792C>T
4	2/M/3	−	Global	−	−	+ (*pes planus*)	−	−	−	+ Wheelchair (10)	−	− (8)	*DYNC1H1* c.751C>T
5	0/F/47	+ (son)	Quadriceps	−	−	+ (achilles, knee and hip retractions, *pes equinovarus*)	−	−	−	+ Wheelchair (49)	+	− (46)	*DYNC1H1* c.2327C>T
6	0/M/21	−	Quadriceps	−	−	+ (achilles and knee restraction, *pes cavus*)	+ (R)	−	+	− (26)	−	− (21)	*DYNC1H1* c.596A>C
7	11/F/11	−	Global	−	−	+ (scoliosis, *pes cavus*)	−	+ (brisk achilles reflexes)	−	− (18)	NA	+ (0)	*BICD2* c.1922T>C
8	10/F/39	−	Psoas	−	−	+ (scoliosis, achilles retraction)	−	−	−	+ Walking stick (50)	−	− (0)	*BICD2* c.2042C>T
9	27/M/33	−	Quadriceps	−	−	+ (*pes cavus*)	−	−	−	− (57)	+	− (17)	*BICD2* c.380A>G
10	14/F/19	−	Psoas	+ (prox.)	+	+ (scoliosis, *pes planovalgus*)	+ (P)	−	+	− (26)	−	− (5)	*ASAH1* c.77C>G and c.125 + 1G>A
11	11/F/12	−	Quadriceps	+ (dist.)	−	+ (*pes cavus*)	−	−	−	+ Wheelchair (23)	−	+ (2)	−
12	38/F/41	−	Global	−	−	+ (scoliosis)	−	−	−	− (59)	+	+ (0)	−
13	18/F/24	−	Psoas	−	+	+ (scoliosis)		+ (brisk achilles and patellar reflexes)	−	+ Walker (61)	−	+ (6)	−
14	52/F/53	−	Global	+ (prox.)	+	−	−	−	−	− (53)	−	− (1)	−
15	1/F/37	− (HSP in son)	Psoas	+ (prox)	−	−	+ (P)	−	−	− (43)	+	− (22)	−
16	30/F/38	+ (sister)	Psoas	−	−	−	−	−	−	− (41)	+	− (8)	−
17	22/F/27	−	Global	+ (dist.)	+	−	+ (R and P)	+ (brisk achilles and patellar reflexes)	−	− (27)	−	− (5)	−
18	22/F/26	−	Quadriceps	−	−	−	−	−	−	− (31)	+	+ (4)	−
19	36/F/49	−	Quadriceps	+ (prox)	+	−	−	−	−	− (55)	+	+ (12)	−
20	53/F/59	−	Psoas	+ (PD)	−	+ (scoliosis, *pes cavus*)	−	−	−	− (59)	+	− (7)	−
21	29/F/50	−	Quadriceps	+ (PD)	+	+	+ (P)	−	+	+ Walker (57)	+	− (28)	−
22	27/M/27	−	Global	−	+	−	+ (P)	−	−	+ Wheelchair (73)	NA	− (46)	−
23	28/F/33	−	Global	−	−	−	−	−	−	− (33)	−	− (5)	−
24	22/F/43	−	Psoas	−	−	−	−	−	−	− (46)	+	− (21)	−

*F* female, *M* male, *SMA* Spinal muscular atrophy, *NA* not available, *CK* creatine kinase, *EMG* needle electromyography, *prox.* proximal, *dist.* distal, *PD* proximo-distal, *R* resting, *P* postural, *HSP* hereditary spastic paraplegia.

The precise clinical description of the cohort is available in Table 3. Tremor was mainly postural tremor, observed in five patients, while resting tremor was observed in only two patients. Pyramidal signs (i.e. brisk reflexes in the lower limbs) were found in only three patients, including one with a *BICD2* variant. No cerebellar syndrome was found. Data regarding walking aid were not compared between groups because of heterogenous follow-up durations between individuals. In the group of patients with identified variants, 3/10 had a walking aid at the last exam, two of whom were wheelchair-bound (both with *DYNC1H1* variants). Interestingly, four of the six patients with *DYNC1H1* disease-causing variants presented with neurodevelopmental disorders or cognitive impairments. Patient 4 was diagnosed with attention deficit hyperactivity disorder (ADHD), and patient 1 presented learning difficulties during childhood. Patients 5 and 6 were diagnosed with impaired executive functions, and patient 5 had added attention and visual deficits. Brain MRI was only available for patient 6 and was normal.

All patients had motor neuropathy on EMG with a neurogenic pattern on needle examination, and five patients had decreased CMAP (three with identified variant, two without). Motor conduction velocities and SNAP amplitudes were normal in all patients.

## Discussion

This study provides an overview of the French landscape of non-5q-SMA, by reporting a proven genetic cause in ten patients from nine families (around 40% of index cases), due to variants in three genes: *DYNC1H1*, *BICD2*, and *ASAH1*. Thus, 60% of index cases remained undiagnosed. *DYNC1H1* mutations, found in five index cases herein, were associated with SMA for the first time by Harms et al. in 2012 [13]. *DYNC1H1* encodes the heavy chains of dynein proteins, which play an important role in retrograde axonal transport [13]. The second most common gene found in this cohort was *BICD2*, with variants found in three patients. Mutations in *BICD2* were first associated with the SMA phenotype in 2012 [14]. The BICD2 protein recruits through its N-terminal domain the dynein protein and promotes the interaction between dynein and dynactin, thus interacting with axonal transport [14]. Patients with either *DYNC1H1* or *BICD2* mutations classically present with a proximal lower limb predominant weakness, associated with frequent osteoarticular deformities such as clubfoot and contractures, and possible scapular winging, which is in accordance with the phenotypes of the patients presented herein [15]. In the present cohort, most of the patients with *DYNC1H1* mutations had cognitive impairments or neurodevelopmental disorders, as previously described [15]. Upper motor neuron signs have been reported in *BICD2*-related SMA, as noticed in one of the patients herein [16]. Finally, one last patient had biallelic variants in *ASAH1* gene, with an autosomal recessive transmission. All the other variants of the cohort were dominant. This woman, whose lower limb weakness started at 14 years old, presented a pure motor phenotype without epileptic myoclonic seizures, contrary to the classical phenotype associated with *ASAH1* mutations named SMA with progressive myoclonic epilepsy (SMA-PME) [17]. This woman has already been reported in a previous case-report [12]. Overall, this cohort shows that two genes, *DYNC1H1* and *BICD2*, account for nearly 40% of the non-5q-SMA French patients. These data also confirm that *CHCHD10* mutations, which are frequent in the Finnish population of non-5q proximal SMA patients, and the previously described *TRPV4* and *VAPB* mutations are not a major feature in the French population [18, 19].

A major issue in hereditary neuropathy is the identification and interpretation of VUS. Indeed, misclassification of a variant can lead to clinical or legal consequences, especially concerning genetic counseling. Here, by adding new reports, we give new arguments for the pathogenicity of three variants initially classified as VUS or with conflicting interpretations in the ClinVar database [10]; one in the *DYNC1H1* gene (c.751C>T) and two in the *BICD2* gene (c.2042C>T and c.1922T>C). Among the three variants not previously described that we report here, two were classified as likely-pathogenic according to the ACMG criteria (c.596A>C and c.1427T>C in *DYNC1H1*) while the last one (c.380A>G in *BICD2*) was classified as a VUS. This variant was predicted to be pathogenic by three in silico algorithms. However, a genetic analysis of *BICD2* in the patient’s parents would certainly help determine the inheritance of this variant and thus, confirm or refute its pathogenicity.

Herein, most variants of the index cases (7/9) were identified through an NGS panel. Only the biallelic variants in *ASAH1* and the c.751C>T variant of *DYNC1H1* were identified by WES and WGS, respectively, performed directly without previous NGS panel testing. WGS was performed in the context of the “AURAGEN” project in patient 4 because a myopathy was initially suspected based on clinical examination [20]. The resulting variant identified in *DYNC1H1* could have been identified by the NGS panel since the latter includes this gene. Conversely, the *ASAH1* variant could not have been detected by the NGS panel, because this gene is currently not included. We thus recommend updating the gene panels of inherited peripheral neuropathies by including *ASAH1*. When WES was performed after the NGS panel in the patients without an identified causative variant, no further variants were identified. The first explanation could be the high number of genes in the NGS panel used, which includes most genes already associated with non-5q-SMA (*DYNC1H1, BICD2, CHCHD10, TRPV4, VAPB…)*. The diagnostic effectiveness of WES or WGS performed after an NGS panel logically depends on the type and number of genes included in the panel. In a previous study of non-5q-SMA and axonal Charcot-Marie-Tooth (CMT2) patients, the diagnostic contribution of WES compared to an NGS panel dropped from 53% to 11% when the initial NGS panel was enriched (62 to 479 genes) [21]. Secondly, in the present cohort, the analysis was performed on sporadic cases or only on the index cases of families (except for patients 2 and 3). WES is known to be more efficient when performed on parent-child trios or on several affected members of the same family. In a previous study, trio WES was able to solve seven of the 25 individuals with a suspected diagnosis of non-5q-SMA [8]. Moreover, WES has already led to the detection of new genes in hereditary peripheral neuropathies, such as *NEFH* [22].

Performing an NGS panel or a WES as a first genetic testing can be discussed. Compared to NGS panel, WES has certain limitations: (i) a suboptimal gene coverage entailing that pathogenic variants could be missed; however in a study in non-5q-SMA/CMT2, none of the detected variants by NGS panel was inside the low covered regions by WES [21]; (ii) a large volume of data are created, thus their interpretation is more time consuming; (iii) the necessity to apply filters due to the huge number of variants found by WES, which could lead to the exclusion of some variants potentially responsible for the disease, iv) WES is more expensive. However, WES has certain advantages compared to NGS panels, such as the possibility to specifically re-analyze one gene when a patient’s phenotype is changing, or the possibility to retrospectively analyze new genes, not described at the time of the initial genetic analysis. Moreover, WES is a very good option when the phenotype is complex, and when several patients in one family are symptomatic. Finally, some of the aforementioned limitations of WES, especially the large number of variants generated and its cost, can be counterbalanced by the use of virtual gene panels, which have been shown to be efficient in reducing the interpretation workload while maintaining good diagnostic rates [23]. Taken together, these observations suggest that in non-5q-SMA patients, a large NGS panel should be the first choice, before performing WES or WGS.

In hereditary peripheral neuropathies, WES and NGS panels have been shown to be effective in detecting pathogenic copy number variations [24]. However, a common limitation of WES and NGS panels is the inability to detect long expansions. Thus, some diseases require specific research, such as Spinal and Bulbar Muscular Atrophy/Kennedy disease characterized by a CAG expansion in the *AR* gene [25]. In such cases, WGS, which is increasingly available, would show clear advantages as it allows the detection of deep intronic mutations and expansions. For instance, WGS allowed the identification of a 10-bp repeat biallelic expansion in the *VWA1* gene as a cause of non-length-dependent hereditary motor neuropathy [26]. In the present study, this genetic abnormality would have been detected by WES because the expansion region was covered with a mean depth of 85, and the number of pathogenic repeats in *VWA1* is known not to be high. WGS is also more effective in detecting deep intronic mutations. Indeed, some hereditary neuropathies could be the consequence of abnormalities of the non-coding genome [27], and one intronic variant in *SOD1* (c.358-10T>G) has been shown to be responsible for ALS with an SMA-like pattern [28]. However, despite good coverage, this particular variant was not identified by WES in the present study.

In regards to the factors that could be predictive of a positive genetic test in patients with suspected hereditary neuropathies, the present study is consistent with previous ones: the age at onset seems to be the more relevant [24, 29]. Moreover, a disease-related variant was identified in three-quarters of the patients for whom symptoms began before the age of 18 years. While a family history of neuropathy was associated with a positive test by *Cortese et al*., such a significant association was not found herein, probably due to a lack of power secondary to the small size of the present cohort [24]. Even if the difference was not significant, osteoarticular deformities and a pure lower limb phenotype were more frequent in the patients with identified causative variants. This could be explained by the high frequency of *DYNC1H1* and *BICD2* variants, in which these abnormalities are frequent [14, 15].

In conclusion, this study supports the realization of a large targeted NGS panel in patients with non-5q-SMA as first genetic testing, while WES, especially when several members of the same family are affected and/or when trio analyses are possible, or WGS should be used as second-line tests.

### Supplementary information

Supplementary Materials

## Data Availability

Anonymized data will be shared upon request from any qualified investigator for purposes of replicating procedures and results. The variants reported in this article have been submitted to an established database (ClinVar, accession numbers SCV003836494 - SCV003836501).
