# Supplementary material for: Genetic characterization of non-5q proximal spinal muscular atrophy in a French cohort: the place of whole exome sequencing
Source: Eur J Hum Genet. 2023 Jun 19;32(1):37–43. doi: 10.1038/s41431-023-01407-8 (PMC10772122; doi:10.1038/s41431-023-01407-8)
Supplement: Supplementary file 1 — Supplementary Materials [file 41431_2023_1407_MOESM1_ESM.pdf]

Supplementary Table 1: List of genes included in the next-generation sequencing panel

**Genes included**
AARS1, AIFM1, ARHGEF10, ATL1, ATL3, ATP1A1, ATP7A, BAG3, BICD2, BSCL2, CCT5, CHCHD10, COX6A1, CTDP1, DCAF8, DCTN1, DHTKD1, DNAJB2, DNM2, DNMT1, DST, DYNC1H1, EGR2, ELP1, FBLN5, FBXO38, FGD4, FIG4, GAN, GARS1, GBF1, GPAD1, GJB1, GNB4, HARS1, HINT1, HK1, HSPB1, HSPB3, HSPB8, IFRD1, IGHMBP2, ITPR3, INF2, JPH1, KARS1, KIF1A, KIF1B, KIF5A, LITAF, LMNA, LRSAM1, MARS1, MFN2, MME, MORC2, MPZ, MTMR2, MYH14, NAGLU, NDRG1, NEFH, NEFL, NGF, NTRK1, PDK3, PLEKHG5, PMP2, PMP22, PNKP, PRDM12, PRPS1, PRX, RAB7A, REEP1, RETREG1, SBF1, SBF2, SCN9A, SCN10A, SCN11A, SEPT9, SETX, SH3TC2, SIGMAR1, SLC12A6, SLC5A7, SORD, SPTLC1, SPTLC2, SURFI, SYT2, TFG, TRIM2, TRPV4, TTR, UBQLN2, VAPB, VCP, VRK1, WARS1, WNK1, YARS1

Supplementary Table 2: List of genes individually checked in whole exome sequencing.

**non-5q-SMA associated genes***	**ALS genes****
ADPRS, ARSA, ASAH1, ASCC1, AGTPBP1, BICD2, C12orf65, CHCHD10, DGUOK, DNM1L, DYNC1H1, ECHS1D, ERBB3, EXOSC3, EXOSC8, FDXR, GLE1, GM2A, HEXA, HEXB, IGHMBP2, LAS1L, LMNA, LYST, MORC2, MPV17, NDUFS6, PDXK, POLG, RRM2B, SCAR4, SCO2, SCP2, SEPSECS, SETX, SLC25A6, SLC52A2, SLC52A3, SMN1, SORD, SPG11, TFG, TK2, TRIP4, TRPV4, TSEN54, TTN, UBA1, UBE1, UBQLN1, VAPB, VRK1	*ANXA11, CCNF, CHCHD10, CHMP2B, DAO, DCTN1, DNAJC7, EPHA4, ERBB4, EWSR1, FIG4, FUS, GLE1, HNRNPA1, KIF5A, MAPT, MATR3, NEFH, NEK1, OPTN, PFN1, PRPH, SOD1, SETX, SPTLC1, SQSTM1, SS18L1, TAF15, TARDBP, TBK1, TUBA4A, UBQLN2, VAPB, VCP*

* Inspired by Pinto *et al.* and Keller *et al.* (7, 8)

** Inspired by Grassano *et al.* (9)

Supplementary Table 3: Prediction of pathogenicity of the three newly identified variants, according to three *in silico* predictors of variant pathogenicity.

**Gene**	***DYNC1H1***		***BICD2***
**Variant**	**c.596 A>C (p.Asn199Thr)**	**c.1427 T>C (p.Leu476Pro)**	**c.380 A>G (p.Glu127Arg)**
MutationTester	Disease-causing	Disease-causing	Disease-causing
PolyPhen2	Probably damaging	Probably damaging	Possibly damaging
SIFT	Affects protein function	Affects protein function	Affects protein function

SIFT: Sorting Intolerant From Tolerant.

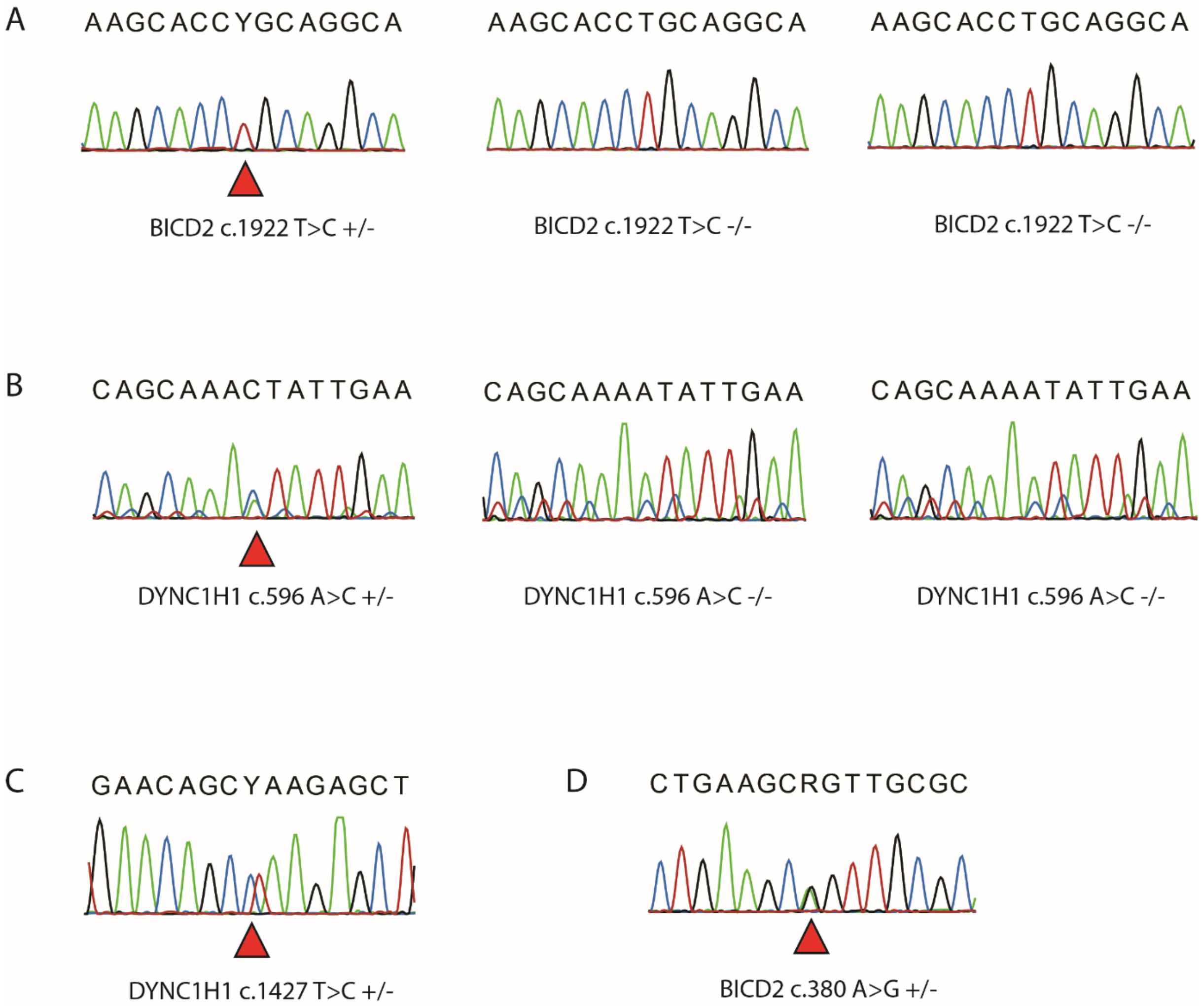

Supplementary Figure 1: Sanger sequencing of the de novo and/or not previously reported variants, identified by gene panel analysis.

A: Sanger sequencing of the c.1922T>C variant in *BICD2* in the patient (left, red triangle), which is absent in the parents (middle and right), B: Sanger sequencing of the c.596A>C variant in *DYNC1H1* in the patient (left, red triangle), which is absent in the parents (middle and right), C: Sanger sequencing of the c.1427T>C in *DYNC1H1* (red triangle); Sanger sequencing in the parents was not performed, D: Sanger sequencing of the c.380A>G in *BICD2* in the patient (red triangle); Sanger sequencing in the parents was not performed.
